# Insights into Activation Pathways of Recovered Carbon Black (rCB) from End-of-Life Tires (ELTs) by Potassium-Containing Agents

**DOI:** 10.1021/acsomega.4c03160

**Published:** 2024-06-13

**Authors:** Bartosz Dziejarski, Robin Faust, Jarosław Serafin, Renata Krzyżyńska, Klas Andersson, Pavleta Knutsson

**Affiliations:** † Department of Chemistry and Chemical Engineering, Division of Energy and Materials, Chalmers University of Technology, SE-412 96 Gothenburg, Sweden; ‡ Department of Space, Earth and Environment, Division of Energy Technology, Chalmers University of Technology, SE-412 96 Gothenburg, Sweden; § Faculty of Environmental Engineering, Wroclaw University of Science and Technology, 50-370 Wroclaw, Poland; ∥ Department of Inorganic and Organic Chemistry, University of Barcelona, Martí i Franquès, 1-11, 08028 Barcelona, Spain; ⊥ Department of Chemical Engineering, University of Utah, Salt Lake City, Utah 84112, United States

## Abstract

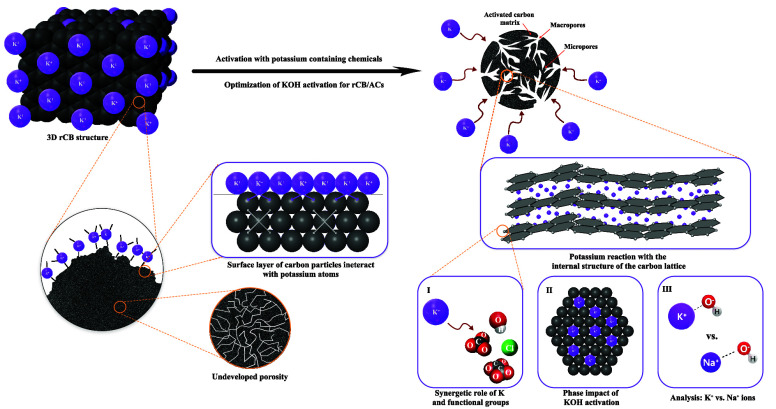

This study explores the conversion of recovered carbon black (rCB) from end-of-life tires (ELTs) into activated carbons (ACs) using potassium-based activators, targeting enhanced textural properties development. The research focuses on the interaction between potassium and rCB, with the aim of understanding the underlying mechanisms of rCB activation. The study investigates several parameters of KOH activation, including the KOH/rCB mass ratio (1:3 to 1:6), activation temperatures (700–900 °C), activation time (1–4 h), and heating rate (5–13 °C/min). It also assesses the effects of different potassium salts (KCl, K_2_CO_3_, CH_3_COOK, and K_2_C_2_O_4_) on porosity and surface characteristics of the rCB/ACs. Furthermore, the role of the physical state of KOH as an activator (solid and gas–solid) was examined, alongside a comparative analysis with NaOH to evaluate the distinct effects of potassium and sodium ions. Optimal conditions were identified at an 800 °C activation temperature, a 7 °C/min heating rate, a 1:5 KOH/rCB ratio, and a 4 h activation period. X-ray diffraction analysis showed the formation of several K-phases, such as K_2_CO_3_, K_2_CO_3_·1.5H_2_O, K_4_(CO_3_)_2_·(H_2_O)_3_, KHCO_3_, and K_2_O. The effectiveness of the potassium salts was ranked as follows: KOH > K_2_C_2_O_4_ > CH_3_COOK > K_2_CO_3_ > KCl, with KOH emerging as the most effective. Notably, the gas–solid reaction of KOH/rCB was indicated as a contributor to the activation process. Additionally, it was concluded that the role of KOH in enhancing the textural properties of rCB was primarily due to the interaction of K^+^ ions with the graphite-like structure of rCB, compared to the effects observed with NaOH. This research introduces novel insights into the specific roles of different potassium salts and KOH activation conditions in optimizing the textural characteristics of rCB/ACs.

## 1 Introduction

Despite the European Union’s prohibition on the accumulation of end-of-life tires (ELTs) in landfills since 2009, challenges in enforcement and inadequate infrastructure persist. The number of vehicles increases annually, rising from 1.2 billion since 2014, and it is anticipated that the worldwide count will exceed 1.6 billion by 2024. This growth highlights the escalating challenge of managing the environmental impact of ELTs waste flow. Nowadays, more than 50% of ELTs are disposed of without undergoing any form of treatment, and it is projected that the annual number of discarded tires will reach 1.2 billion by 2030.^1^ ELTs can be a source of environmental contamination, which is a result of the release of hazardous substances into soil and water systems during their degradation.^2^ Furthermore, tires contain natural (∼14–30%) and synthetic (∼14–27%) rubber, fabric/fillers/accelerators antiozonants (∼10–17%), and a substantial amount of carbon in the form of carbon black (∼20–28%) and steel wire (∼14%).^3^ Due to ELTs relatively constant chemical content and their general availability, they are a well-suited resource for the production of activated carbons (ACs) for which the presence of a high carbon content is a prerequisite. The pyrolysis technique has been recognized as the most effective method for recycling ELTs, with no economically viable alternative currently available. Pyrolysis facilitates the breakdown of the organic constituents included in tires, resulting in the formation of pyrolytic char that contains a high proportion of recovered carbon black (rCB) as well as residues of carbonization, as a solid raw material.^4^ Therefore, a purification stage for char is essential to obtain high-value rCB with a large carbon content (up to 95%) and to maximize the yield of ACs. In view of this, the conversion of ELTs into rCB/ACs provides a sustainable approach not only to managing waste flows but also for producing value-added products.

Activated carbons are primarily produced by the carbonization and activation of carbon precursors with little ash content, including solid fossil fuels (such as hard coal, brown coal, or peat), biomass (lignin, wood, fruit seeds, nut shells, or bamboo), and polymers (poly(ethylene terephthalate) (PET) bottles, packaging wastes, or epoxy resins).^5−7^ The synthesis of ACs from carbon-based materials involves a sequence of crucial procedures referred to as pretreatment and modification. Pretreatment techniques, including washing, drying, and thermal treatment, are essential for purifying carbonaceous precursors and enhancing their reactivity. These steps facilitate the removal of impurities and prepare the surface of the precursor for subsequent activation.^8−10^ As a result, ACs are mainly composed of elemental carbon in an amorphous state and fine-crystalline graphite. Currently available commercial ACs remain costly owing to their reliance on nonrenewable precursors, which increases the demand for ACs that can be produced from a wide range of low-cost raw materials. The benefits of ACs are strictly related to their distinct physicochemical properties like a large specific surface area, a well-defined network of pores, significant mechanical strength, and abundant functional groups with various oxygen-containing compounds that can be tailored for the specific application by activation processes.^11,12^

An activation process is necessary for achieving the desired properties of ACs.^13,14^ Activation may be accomplished by several methods, including chemical or physical processes. Chemical activation is more favorable since it requires lower temperature, has higher efficiency, and requires shorter processing time compared to a physical one.^15^ It involves treatment with oxidizing acids (HNO_3_, H_2_SO_4_, H_3_PO_4_), salt solutions (ZnCl_2_, MgCl_2_), or alkalis (KOH, NaOH, K_2_CO_3_). Different researchers^16−19^ proved that the choice of activating agent such as H_2_SO_4_, H_3_PO_4_, HCl, NaOH, and KOH further modulates the properties of the ACs. Each activator operates through distinct mechanisms, impacting pore development and surface chemistry in unique ways. Furthermore, the activation parameters along with the carbonization process play a pivotal role in shaping the structure and characteristics of the resulting activated carbons.^20^ Factors such as temperature, heating rate, residence time, mass ratio of precursor:activator, and ambient atmosphere exert significant influence on the final product. While elevated temperatures generally lead to higher carbon content and surface area, they also affect pore size distribution and chemical composition.

Among the various activators, potassium-containing agents such as KOH and K_2_CO_3_ are particularly favored due to their strong alkalinity, reactivity with carbon, ability to widen pore size distributions, increase total pore volume, and create a highly porous structure, especially enhancing microporosity.^21−26^ Furthermore, potassium tends to enhance the activation kinetics, leading to a more efficient thermochemical conversion.^27,28^ Previous research on KOH and K_2_CO_3_ has suggested different chemical reactions to explain potassium effect.^8,29,30^ Widely, it is assumed that KOH possesses a tendency to engage with carbon atoms, therefore stimulating the dehydrogenation process (removal of hydrogen atoms from the carbon precursor material) that ultimately results in the formation of a carbon structure characterized by a well-defined porous morphology.^31−33^ While the efficacy of KOH and K_2_CO_3_ is well-documented, the exploration of other potassium-based activators in the production of ACs prompts a closer investigation into the underlying mechanisms.^34−37^ This aims to reveal the different interactions of potassium and specific ionic groups and their effects on the AC production process, especially when using diverse precursors. Furthermore, each of these agents interacts with AC precursors in ways that could potentially optimize or enhance specific properties of the resulting ACs, justifying their investigation alongside traditional activators, such as KOH and K_2_CO_3_.

Initial investigations have explored the viability of ELT char during chemical activation to optimize textural properties (specific surface area, total pore volume, and microporosity).^38−43^ The main goal of these investigations has been to examine the effectiveness of potassium hydroxide (KOH), potassium carbonate (K_2_CO_3_), phosphoric acid (H_3_PO_4_), zinc chloride (ZnCl_2_), sodium hydroxide (NaOH), and sodium carbonate (Na_2_CO_3_). Among the tested activating agents, the application of KOH has shown significant enhancements in specific surface area (180–820 m^2^/g), porosity development (0.28–1.32 cm^3^/g), and tailored pore size distribution of char/ACs compared to those of other chemicals. Al-Rahbi and Williams^39^ claimed that tires char-based ACs produced by the KOH activation with a mass ratio of 1:3 at a temperature of 900 °C had a more prominent porosity compared to those treated with alternative alkali activating agents, including K_2_CO_3_, NaOH, and Na_2_CO_3_. Hofman and Pietrzak^40^ observed that the KOH has a notable positive influence on the creation of ACs from waste tires char that exhibit a highly developed porous structure, mostly composed of micropores, when activated at a temperature of 800 °C. Furthermore, the research performed by Nieto-Márquez et al.^38^ proved that the rise in the proportion of the KOH to waste tires ratio subsequently resulted in a proportional augmentation in surface area, and total pore volume of ACs.

While these findings offer encouraging insights, their scope is limited, focusing solely on examining the dependence of temperature (550–900 °C) and char/activator mass ratio (1:0.5 to 1:4). It did not thoroughly examine how K-containing agents interact with char carbon atoms. Additionally, the studies neglected the impact of other critical activation factors (heating rate or activation time), the formation of specific chemical phases during the thermal process, and the effects of KOH phase transitions. This focus highlights a significant gap, underscoring the need for in-depth research into rCB activation mechanisms. On the other hand, in the field of alkali-enhanced gasification and combustion, the practice of converting solid fuels and waste materials with alkali metals is well-established due to their recognized catalytic role in promoting the efficiency of the overall process.^44−46^ Most proposed mechanisms involve the presence of potassium in a metallic state, produced at high temperatures (800–900 °C). Metallic K is capable of intercalating into the carbon framework, creating intercalation compounds that leads to the expansion of graphitic lattice.^47,48^

This work aims to provide insights into the mechanism during the activation of rCB to ACs by potassium-containing activators. The main goal is to improve the textural properties of the rCB/ACs. KOH was selected as the baseline chemical to examine the effect of the KOH/rCB mass ratio, temperature, activation time, and heating rate influence on the characteristics of produced rCB/ACs, as suggested in the literature. It is recognized that different AC precursors might require tailored activation parameters to optimally develop their porous structures and surface characteristics. The research thoroughly explores these interactions, examining how changes in the KOH activation parameters impact rCB. Then, for the assumed standard parameters, different potassium salts, including KCl, K_2_CO_3_, CH_3_COOK, and K_2_C_2_O_4_·H_2_O, were employed to investigate the potential synergistic role of K^+^ with specific functional groups (OH^–^, Cl^–^, CO_3_^2–^, COOK^–^, C_2_O_4_^2–^) in the development of porosity and their effect on the rCB/AC surface. Additionally, the impact of the state of the activating agent (solid-state or gaseous-state reaction) and a comparative analysis with NaOH (Na^+^ vs K^+^) were explored. Furthermore, it is crucial to test industrially produced material to adapt laboratory findings for industrial-scale production, ensuring that the process is viable and effective on a larger scale.

Herein, the key objectives of this work were to fill this gap in knowledge by (1) revealing the optimal conditions for the rCB activation using K-based activators; (2) establishing the responsible factors in the conversion of rCB to ACs using potassium alkalis for porosity development; and (3) uncovering the generic mechanism driving rCB activation to efficiently convert the ELTs into ACs. Through laboratory experiments and material characterization, we provide potential activation pathways of rCB conversion to ACs with emphasis put on interactions with potassium. Understanding the potassium interaction with the rCB surface and optimizing the activation of potassium-containing agents of rCB has important implications as it can improve the production of ACs, aiding sorption application, and promote sustainability by using waste materials like ELTs.

## 2 Experimental Section

### 2.1 Chemicals and Materials

The reagents used for the synthesis of rCB/ACs were KOH, K_2_CO_3_, KCl, CH_3_COOK, K_2_C_2_O_4_·H_2_O, and NaOH with a purity of 97%. The activated carbon precursor employed in this study was tailor-made rCB obtained from a company in Poland. The production process involved treating char through devolatilization, milling, and, if necessary, pelleting to reduce the ash content, remove metal impurities, and normalize variations in the particle size. The chemical composition of rCB is notably variable and contingent on the composition of the ELTs subjected to industrial-scale pyrolysis. Carbon stands out as the principal component, ranging from 92% to 99.5 wt %, as shown in Table 1. Additionally, rCB may encompass volatile substances (>0.2 wt % content) and mineral matter (0.5–2 wt % content), including elements like Si, Al, Zn, S, and Ca.

**Table 1:** Chemical Composition of rCB in wt %

sample	C [wt %]	H [wt %]	N [wt %]	S [wt %]	O [wt %]
rCB	92.53	1.16	0.42	0.71	5.18

### 2.2 Preparation of rCB-Based Activated Carbons

**Figure 1:**
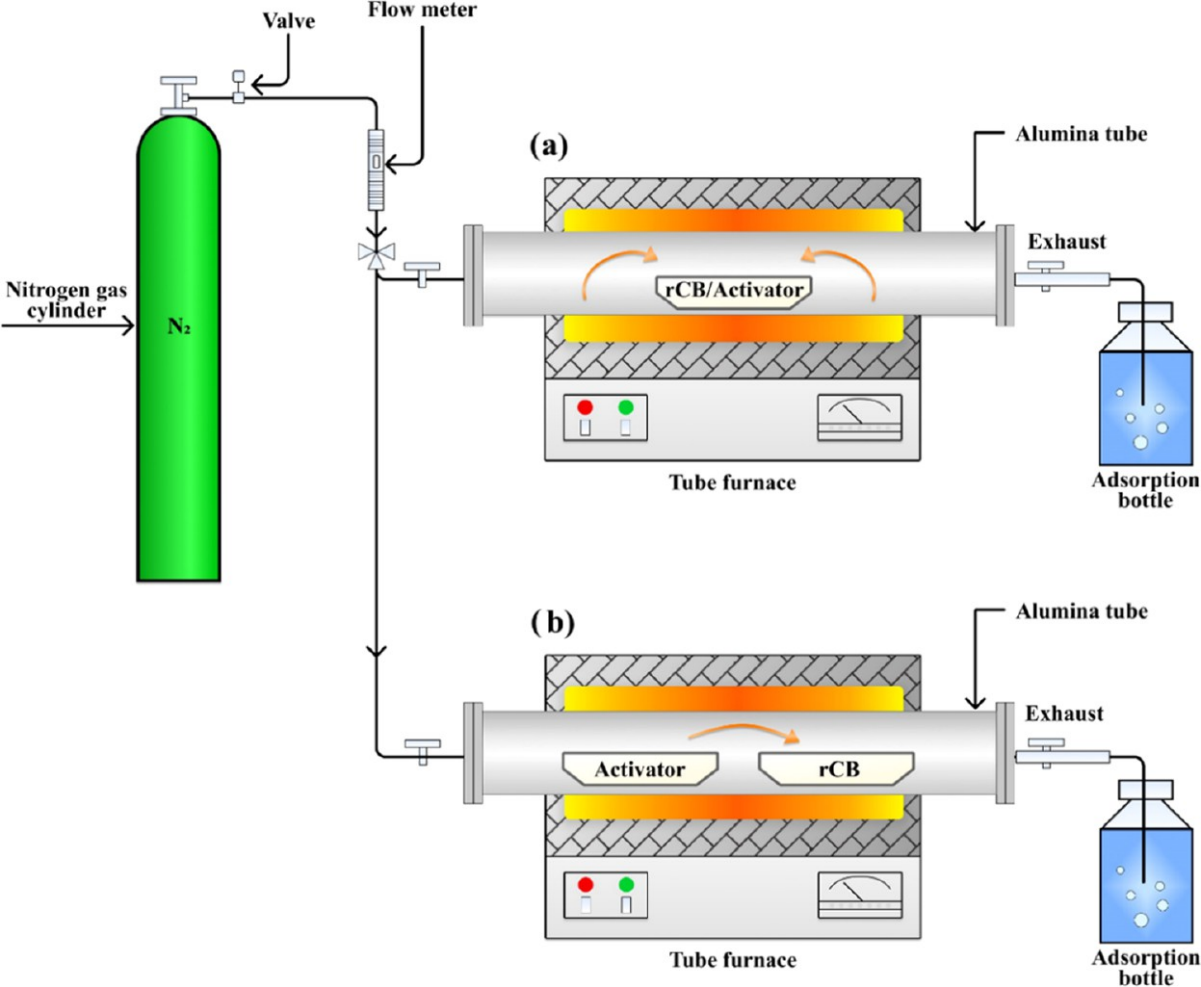
Schematic diagram of the experimental process setup applied for solid/liquid-solid state activation (a) and gas-solid state activation (b).

The methodology employed in this study involves testing several key factors of KOH activation, including the reaction phase, mass ratio, temperature, heating rate, and activation time, as these are known to have a significant impact on the overall process. By systematic examination of these variables, the activation mechanism can be followed and optimized. The materials were exposed to tube furnace conditions. About 8 g of mixture of rCB and activator agent was placed in alumina crucibles and located in the center of the horizontal furnace, as presented in Figure 1. Two types of set rCB activators were tested: gas–solid reactions (activator salt as gas) and solid/liquid–solid (activator salt as solid) reactions. In the case of a gas–solid reaction, the reactants were separated into two crucibles, accordingly. Then, the sample was heated under a continuous flow of N_2_ gas at a rate of 200 cm^3^/min, based on the previous research related with ELTs^38−40,49^ and in accordance with recommendations from the existing literature.^50−52^ The temperature, ranging from 700 to 900 °C, depending on the experimental setup was achieved by a heating rate of 5–13 °C/min and then maintained for a duration of 1–4 h (Table 2). Following that, the sample was exposed to a controlled cooling process, gradually reducing its temperature to correspond with 25 °C at a rate of 5 °C/min.

**Table 2:** Experimental Conditions for Activation Processes

type of activation	heating rate [°C/min]	temperature [°C]	activation time [h]	flow rate of N_2_ [cm^3^/min]	cooling rate [°C/min]
chemical activation	5–13	700–900	1–4	200	5

For the solid-state activation reaction, the rCB was dry mixed in a mortar with specific potassium-containing (KOH, K_2_CO_3_, KCl, CH_3_COOK, and K_2_C_2_O_4_·H_2_O) or sodium-containing (NaOH) activating agents in the solid–solid phase. The mass ratios between the precursor and the chosen salts ranged from 1:3 to 1:6. Following the completion of the activation step under the selected reaction conditions, half of the resultant solid material was rinsed with water until achieving a neutral pH of the washing solution in order to eliminate salt leftovers and evaluate the textural characteristics of the obtained activated carbons. Simultaneously, the other half of the material that was left untreated was used for the investigation of the carbon–potassium reaction mechanism through X-ray diffraction (XRD) analysis and scanning electron microscopy coupled with energy-dispersive X-ray spectroscopy (SEM-EDS). Finally, the rCB/ACs were subsequently dried at a temperature of 110 °C for a duration of 12 h. The samples were labeled using coding: activating agent, carbonization temperature, and residence time during activation (Tables 3 and 4). For example, the notation KOH_1:3_700_3_5 represents a rCB/AC sample that has undergone activation with KOH in the mass ratio 1:3 at 700 °C for 3 h with a heating rate of 5 °C/min. In line with the published literature concerning the reutilization of pyrolytic char to produce ACs, the baseline activation parameters for the study objectives were determined to be 800 °C, 1:4, 3 h, and 5 °C/min. It should be noted that these parameters were selected by assuming the ACs were not further manipulated to optimize their textural properties.

**Table 3:** Overview of rCB/ACs Sample Symbol Sets for Porosity Development

symbol	mass ratio [-]	temperature [°C]	activation time [h]	heating rate [°C/min]
Effect of KOH Activation Temperature				
KOH_1:4_700_3_5	1:4	700	3	5
KOH_1:4_800_3_5^a^	1:4	800	3	5
KOH_1:4_900_3_5	1:4	900	3	5
KOH Activation Time Influence				
KOH_1:4_800_1_5	1:4	800	1	5
KOH_1:4_800_2_5	1:4	800	2	5
KOH_1:4_800_4_5	1:4	800	4	5
Effect of Mass Ratio Between rCB/KOH				
KOH_1:3_800_3_5	1:3	800	3	5
KOH_1:5_800_3_5	1:5	800	3	5
KOH_1:6_800_3_5	1:6	800	3	5
Effect of Heating Rate				
KOH_1:4_800_3_7	1:4	800	3	7
KOH_1:4_800_3_10	1:4	800	3	10
KOH_1:4_800_3_13	1:4	800	3	13

a The study’s base activation conditions were set at 1:4 ratio, 800 °C, 3 h, and 5 °C/min.

**Table 4:** Description of rCB/ACs Sample Symbol Sets for Research Goals

symbol	activating agent	mass ratio [-]	temperature [°C]	activation time [h]	heating rate [°C/min]
Role of Potassium and Functional Groups in Porosity Development					
CH_3_COOK_1:4_800_3_5	CH_3_CO_2_K	1:4	800	3	5
K_2_CO_3__1:4_800_3_5	K_2_CO_3_	1:4	800	3	5
KCl_1:4_800_3_5	KCl	1:4	800	3	5
K_2_C_2_O_4_ _1:4_800_3_5	K_2_C_2_O_4_	1:4	800	3	5
Phase Impact of KOH Activation					
KOH(vapor)_1:4_800_3_5	KOH	1:4	800	3	5
Potassium Hydroxide (K^+^) vs Sodium Hydroxide (Na^+^)					
NaOH_1:4_800_3_5	NaOH	1:4	800	3	5

### 2.3 Characterization Techniques of Carbon Materials

The analysis of the chemical composition of recovered carbon black was conducted using a FLASH 2000 CHNS Elemental Analyzer from Thermo Fisher Scientific.

The most important qualities of carbon materials are their textural characteristics, such as the surface area, pore volume, and pore size distribution, as well as their crystallographic structure. Thus, these aspects play a crucial role in defining the activation process and are the first parameters to follow in the rCB in order to evaluate a new activation procedure.

The enhancement of textural properties of prepared rCB/ACs was measured by N_2_ adsorption–desorption at 77 K using the fully automated, three-station surface area and porosity analyzer Tristar II 3020 Micromeritics. Prior to conducting adsorption tests, the samples were left in a degassed chamber overnight at a temperature of 250 °C. To calculate the specific surface area of samples (*S*_BET_), the experimental data was fitted to the Brunauer–Emmett–Teller (BET) equation in a relative pressure range of *P*/*P*_0_ from 0.05 to 0.3, as presented below
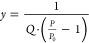
1where *Q* represents the mass of gas adsorbed at a given relative pressure (*P*/*P*_0_), *P* is the pressure of the nitrogen gas used in the adsorption experiment [mmHg], and *P*_0_ is the saturation pressure of nitrogen gas [mmHg].

Additionally, the selection of a proper *P*/*P*_0_ range was based on a modification of the BET theory proposed by Rouquerol et al.^53,54^ to obtain the highest linear relationship in regard to the coefficient of determination (*R*^2^ ∼ 1).

The total pore volume (*V*_TOT_) was determined from the N_2_ adsorption isotherm at a high relative pressure, typically close to unity (0.99 or higher). The examination of micropore volume (*V*_MIC_) within the range of pore diameters from 1.4 to 2 nm and pore size distribution (PSD) was conducted using the density functional theory (DFT) approach, specifically by applying the nonlocal density functional theory (NLDFT) model. Furthermore, the mesopore volume (*V*_MES_) was estimated by subtracting the micropore volume from the total pore volume, utilizing the assumption that mesopores (2–50 nm) constitute the difference between these two volumetric measurements.

The crystallographic structure of the rCB/ACs was examined by X-ray diffraction analysis using a Bruker D8 Discover X-ray diffractometer. The particles were finely powdered before the patterns were recorded. Cu Kα radiation was applied (λ = 0.15418 nm) as the radiation source, and the diffraction patterns were recorded at regular 2θ intervals covering 10–85°. The identification of the relevant phases created during activation was performed using the DIFFRAC.EVA software, with reference to the PDF-4 + 2022 database (ICDD), which was also used for semiquantitative analysis.

Scanning electron microscopy with an energy-dispersive X-ray spectroscopy system was used to analyze the distribution of potassium on recycled rCB/ACs processed with different potassium-containing activators. The analysis was conducted using a JEOL 7800F Prime SEM, operating at 20 kV with a solid-state detector (SSD), utilizing backscattered electrons (BSE). The primary objective of the SEM-EDS analysis was to map the spatial distribution of K on the surfaces of rCB/ACs and to quantitatively evaluate the surface elemental composition. This approach provides insights into the effectiveness of the activation process in incorporating potassium into the carbon structure of the materials.

## 3 Results and Discussion

### 3.1 Effect of KOH Activation Parameters on rCB/ACs Textural Properties

**Figure 2:**
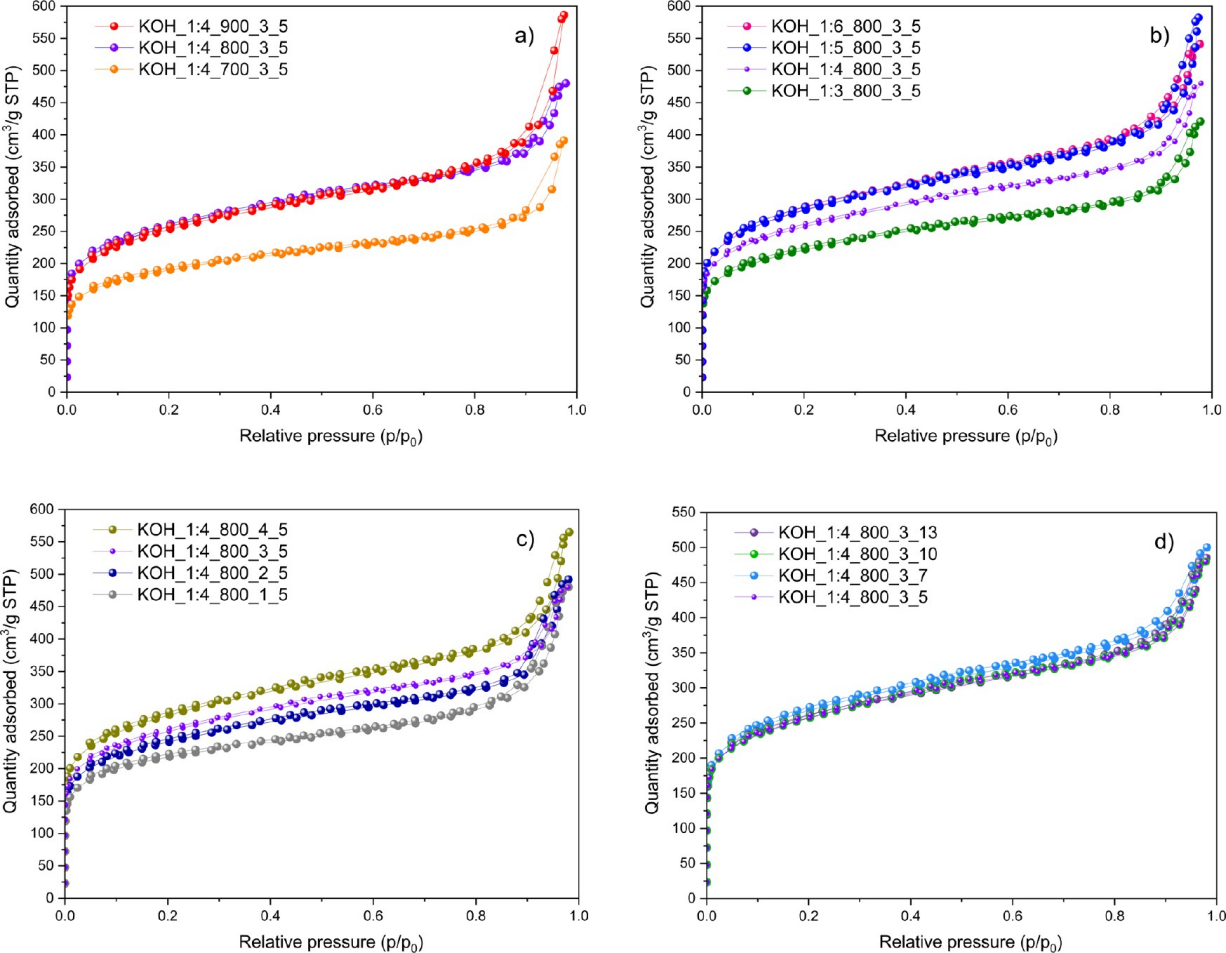
N_2_ adsorption–desorption isotherm curves at 77 K of the rCB/ACs in relation to the activation effect of temperature (a), mass ratio (b), time (c), and heating rate (d).

The optimization of KOH activation parameters, including the temperature (Figure 2a), mass ratio (Figure 2b), residence time (Figure 2c), and heating rate (Figure 2d) emerges as a critical aspect of examining the limitations of the rCB precursor. Based on the obtained N_2_ isotherms for each parameter, the development of the porosity can be followed, which indicates the type of structure of the material that evolves as a result of activation. According to the IUPAC classification, all curves exhibit a mixture of type I and type IV isotherms, indicating a bimodal pore system in the rCB/ACs. This combination suggests the existence of both micropores (<2 nm) and mesopores (2–50 nm).^55^ The presence of micropores was confirmed by the significant N_2_ adsorption found at a *P*/*P*_0_ below 0.05. Furthermore, the hysteresis loops have a nearly narrow, horizontal, and parallel shape throughout a broad range of *P*/*P*_0_ (up to 0.85) with a pointed end, proving the H3-type of isotherm. The H3-type implies that the main pore morphology in all rCB/ACs samples involves slit-shaped pores with a well-developed mesoporous structure.^56^ The desorption isotherm exhibited a hysteresis loop as a result of capillary condensation occurring in mesopores.^57^

The N_2_ adsorption–desorption isotherms were further applied for the determination of the textural properties of all rCB/AC samples along with the related impact of the activation parameters on them, including the specific surface area, total volume of the pores, and mesoporosity and microporosity content (Table 5). The rCB had a very low BET surface area of 55 m^2^/g, lacking micropores, and possessing a limited total pore volume of 0.170 cm^3^/g. The KOH-activated rCB exhibited both micro- and mesoporous characteristics, with the mesopore percentage (determined by the *V*_MES_/*V*_TOT_ ratio) mostly exceeding 60%. These findings demonstrate that the KOH benefits increased porosity with the BET surface of rCB/ACs. Based on Table 5, each examined activation parameter (temperature, mass ratio, activation time, and heating rate) is expected to fall within a certain range to influence the obtained textural properties of the rCB/ACs optimally. Furthermore, through the experiments performed, one can gain an understanding of how each of the varied parameters influences the overall activation mechanism, as shown in Table 3. Ultimately, the specific surface areas of the rCB/ACs in this study exceed those documented by other researchers who activated char formed from ELTs using KOH with the main focus on temperature (550–900 °C) and mass ratio effect (1:0.5–1:4).^38−40^

**Table 5:** Textural Properties Associated with the Produced rCB/ACs by Specific Activation Parameters

sample	BET surface area^a^ [m^2^/g]	total pore volume^b^ [cm^3^/g]	micropore volume^c^ [cm^3^/g]	mesopore volume^c^ [cm^3^/g]	micropore volume/total pore volume [%]
rCB	55 ± 0.8	0.170	-	-	-
Effect of Temperature					
KOH_1:4_900_3_5	915 ± 2.3	0.854	0.288	0.566	39
KOH_1:4_800_3_5	945 ± 5.6	0.743	0.303	0.440	36
KOH_1:4_700_3_5	688 ± 1.8	0.605	0.225	0.380	37
Effect of Mass Ratio Between rCB/ACs					
KOH_1:6_800_3_5	1022 ± 3.8	0.837	0.312	0.525	39
KOH_1:5_800_3_5	1025 ± 2.2	0.901	0.327	0.574	35
KOH_1:3_800_3_5	801 ± 1.8	0.651	0.252	0.399	39
Effect of Activation Time					
KOH_1:4_800_4_5	1028 ± 1.5	0.874	0.332	0.542	38
KOH_1:4_800_2_5	876 ± 3.08	0.727	0.276	0.467	37
KOH_1:4_800_1_5	788 ± 1.3	0.699	0.211	0.488	30
Effect of Heating Rate					
KOH_1:4_800_3_7	970 ± 1.9	0.884	0.314	0.460	41
KOH_1:4_800_3_10	934 ± 2.0	0.774	0.298	0.452	40
KOH_1:4_800_3_13	900 ± 1.8	0.750	0.289	0.595	32

a Brunauer, Emmett, and Teller method using the Rouquerol criteria.

b V_TOT_ calculated by the N_2_ adsorption isotherm at a high relative pressure (∼0.99).

c DFT method by the NLDFT model.

**Figure 3:**
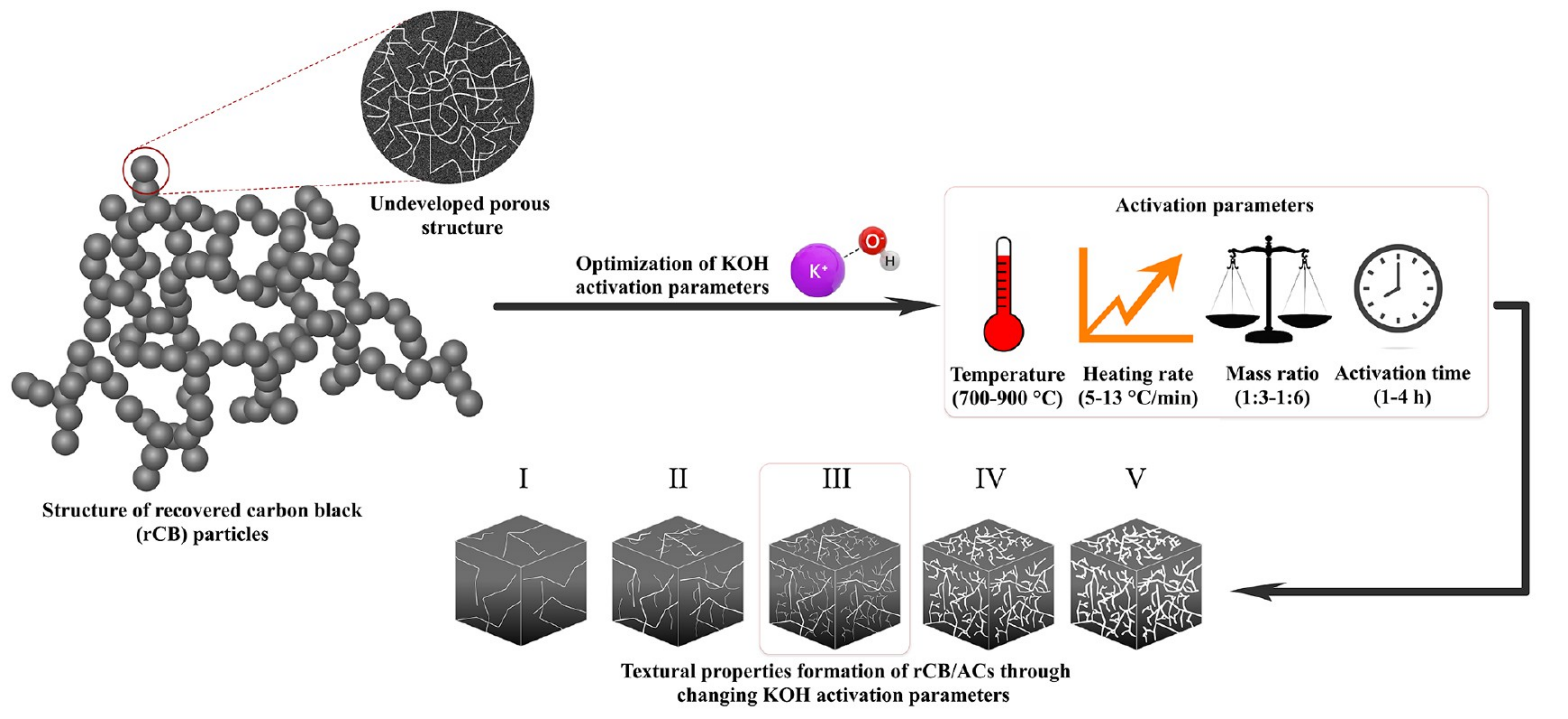
Optimal formation of rCB/ACs textural properties (BET surface area, total pore volume, and microporosity) influenced by KOH activation parameters (III stage, 800 °C, 7 °C/min, 1:5, and 4 h).

As demonstrated previously, the optimal conditions, detailed in Figure 3 highlight the critical role of activation parameters in enhancing the quality and efficiency of rCB/ACs production.

The temperature dependence can be followed when the temperature increased from 700 to 800 °C. As a result of the increased temperature, the KOH activation reaction and diffusion rate with carbon atoms become more intense, leading to the creation of the pore skeleton (0.605–0.743 cm^3^/g), the development of microporosity (0.225–0.303 cm^3^/g), and increase in the specific surface area (688–945 m^2^/g). In the range of 700–800 °C, K^+^ ions can penetrate the carbonized surface more effectively, which leads to the growth of micropores. On the other hand, the temperature dependence observed during the activation of rCB between 800 and 900 °C can be related to the intensified activation of carbonaceous rCB that forms wide interconnected mesopores (0.901 cm^3^/g), which partially degrades the specific area to 915 m^2^/g and decreases the microporous structure to 0.288 cm^3^/g. This observation aligns with the conclusions reported by Serafin et al.^58^ In addition, Hofman and Pietrzak^40^ also proposed that the ideal temperature for the pyrolytic char is 800 °C, with a KOH/rCB ratio of 1:4. Other authors, Al-Rahbi and Williams^39^ indicated that a temperature of 900 °C leads to higher effectiveness in achieving the desired *S*_BET_, *V*_TOT_, and *V*_MIC_, despite the use of a lower mass ratio. That fact potentially implies a significant correlation between these two factors.

Further, when considering the impacts of the rCB/KOH mass ratio (1:6, 1:5, 1:4, and 1:3), the optimized values of *S*_BET_ (1025 m^2^/g), *V*_TOT_ (0.901 cm^3^/g), and *V*_MIC_ (0.901 cm^3^/g) were found to be 1:5. The 1:4 ratio shows a decrease in all properties compared to 1:5, reinforcing the importance of the correct amount of KOH for achieving optimal activation. The performance decreases further at a 1:3 ratio, indicating insufficient activation, while the 1:6 ratio suggests diminishing returns with excess KOH. At ideal levels, KOH efficiently reacts with rCB, ensuring a balanced reaction and the appropriate quantity of KOH for the formation and stabilization of the desired pore structure. However, beyond a certain threshold (≥1:6), excessive KOH can lead to the blockage of pores due to deposition of potassium compounds within them.^59^

For different activation periods (1–4 h), 4 h was the most effective time interval for producing rCB/ACs, resulting in an enhanced surface area (1028 m^2^/g), micropore volume (0.332 cm^3^/g), and a more evenly distributed range of pore sizes (0.874 cm^3^/g). A longer period of time (3–4 h) allows for the complete realization of chemical processes, such as carbonate formation and gasification, required for opening and expanding pores in rCB/ACs. This is directly connected to the overall reaction rate.^60^ Conversely, shorter activation durations (≤3 h) may not provide enough time for these processes to occur fully (788–945 m^2^/g, 0.699–0.743 cm^3^/g).

Upon comparing the heating rates in the range of 5–13 °C/min, it is evident that the best results for *S*_BET_ and developed pore structure, of 970 m^2^/g and 0.774 cm^3^/g, respectively, were attained at 7 °C/min. The consistent trend across different properties indicates a measurable, yet low, impact of the heating rate on rCB/ACs properties. This particular rate achieves an optimal equilibrium between the dispersion of heat and improved kinetics. It is fair to assume that heating rates below 7 °C/min would provide improved contact between the carbon and the molten KOH (>360 °C) before the desired reaction temperature is attained. Moreover, KOH activation leads to the formation of surface oxygen complexes, causing carbon gasification and the release of gases like CO_2_ and CO. The use of a particular heating rate threshold contributes to a more regulated release of gas, which might possibly compensate for the enhanced formation of micropores (0.314 cm^3^/g), as described by Lozano-Castello et al.^61^ Whereas a rate of 5 °C/min may be insufficient, impeding the growth of pores, 10 and 13 °C/min may be excessive, posing a danger of irregular porosity and significant harm to the structure.

**Figure 4:**
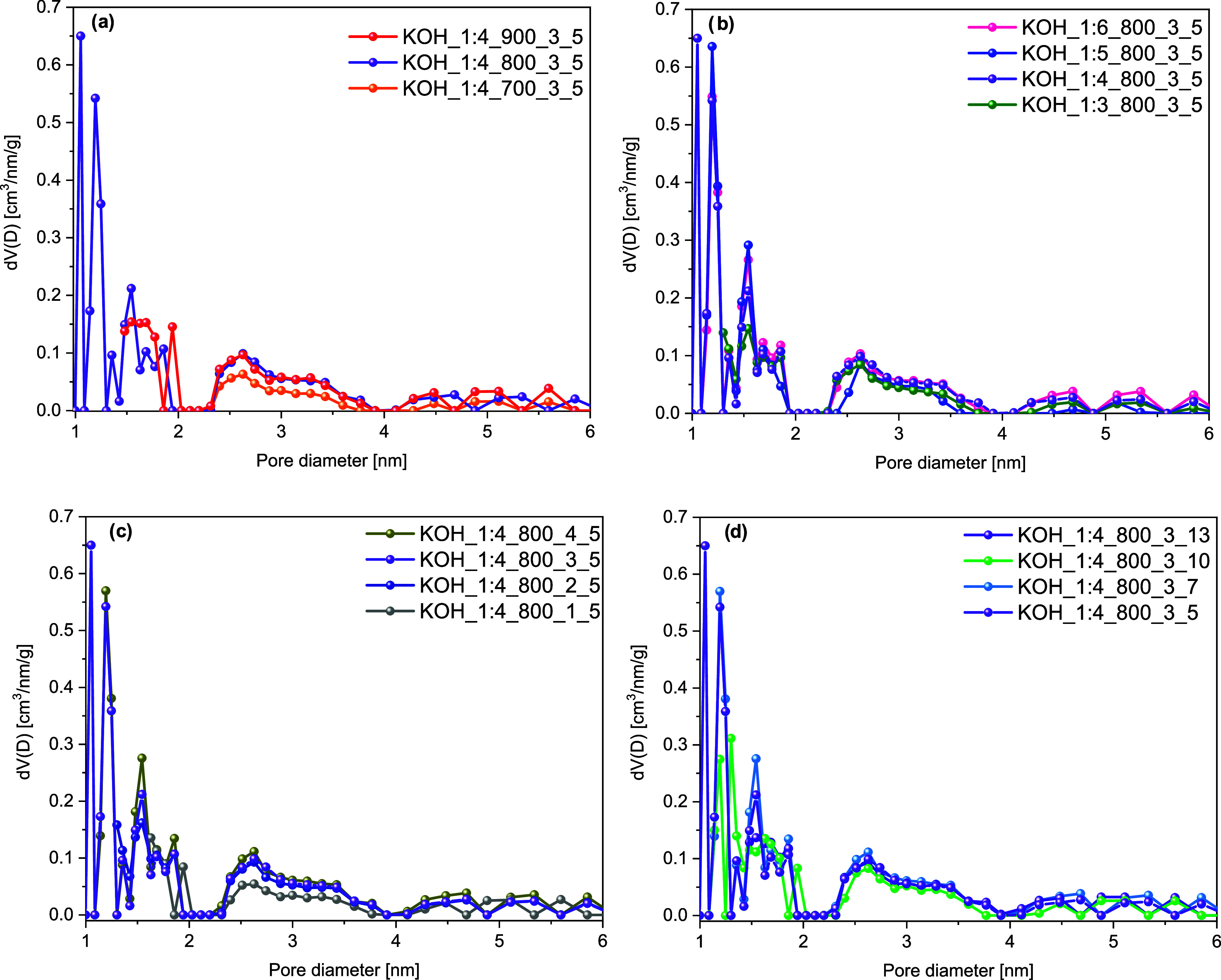
Pore size distribution of the rCB/ACs based on the DFT method of N_2_ adsorption at 77 K on carbon slit pores by the NLDFT model in relation to the activation effect of temperature (a), mass ratio (b), time (c), and heating rate (d).

The pore size distribution of rCB/ACs for each KOH activation parameter is schematically shown in Figure 4a–d, which proves the previous observations. A comparable PSD has been identified for all carbon samples that were analyzed. The application of KOH as an activator led to an enhancement in microporosity that confirmed previous observations (∼30–40% enhancement). The content of micropores in rCB/AC was significantly improved at a temperature of 800 °C, rCB/KOH ratio of 1:5, heating rate of 7 °C/min, and a time of 4 h, illustrating that the pore size was mainly concentrated in three distinct peaks, situated in the ranges of 0.99–1.09, 1.09–1.30, and 1.43–1.63 nm. Furthermore, it was found that the mesopores had a dominating size located within the range of 2.0–13.49 nm.

In continuation of previous discussions, a comparison of activated carbons synthesized from various precursors via KOH activation is presented in Table 6, showing the textural properties, including BET surface area and porosity, as reported in the literature. It is observed that the synthesized rCB/ACs compare favorably with others in terms of *S*_BET_, *V*_TOT_, and *V*_MIC_ values. In the case of *V*_MIC_/*V*_TOT_, a significant distinction exists, which may be related to the fact that rCB features a rigid, spherical, and aggregated structure.^49^ This configuration starkly contrasts with the “model” texture that was earlier identified as optimal for KOH activation, which should ideally develop a high content of micropores.^42^ The closed nature of the rCB structure likely restricts potassium accessibility, thereby hindering its penetration and subsequent reactions within the particles. Nevertheless, limited amounts of potassium can still infiltrate the interior of these particles, fostering porosity through both carbon consumption and intercalation.

**Table 6:** Comparison of Textural Characteristics of ACs Prepared from Various Precursors by KOH Activation

precursor	BET surface area [m^2^/g]	total pore volume [cm^3^/g]	mesopore volume [cm^3^/g]	micropore volume [cm^3^/g]	refs
recovered carbon black (KOH_1:5_800_3_5)	1025	0.90	0.54	0.33	this work
pristine gelatin	1294	0.63	-	-	(62)
starch	714	0.40	-	-	(62)
slash pine	906	0.35	-	-	(63)
bituminous coal	1089	0.50	0.05	0.45	(64)
vine shoots	1032	0.49	0.036	0.35	(65)
garlic peel	947	0.51	0.01	0.50	(66)
coconut shell	1026	0.58	-	-	(67)
petroleum coke	990	0.60	0.05	0.55	(68)
packaging waste	1283	0.69	0.11	0.58	(69)
PET bottles	1165	0.47	0.01	0.46	(70)

### 3.2 Analyzing the Interplay of Factors in the KOH Activation Process

**Figure 5:**
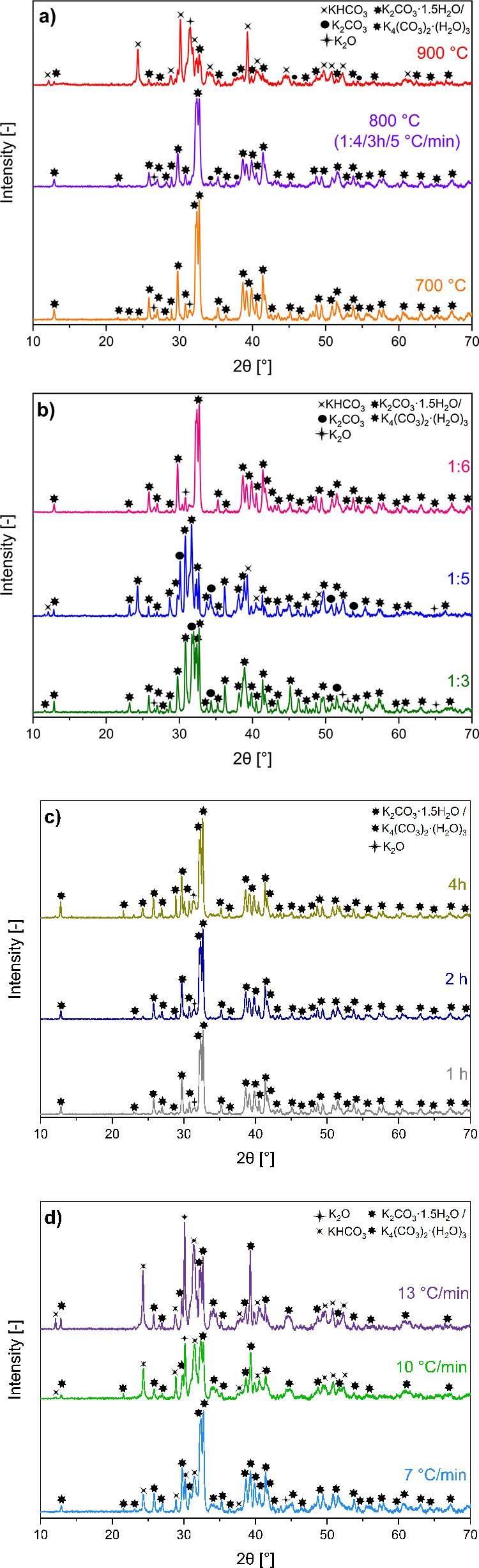
XRD patterns for rCB/ACs before washing treatment related to the effect of KOH activation parameters: temperature (a), mass ratio (b), activation time (c), and heating rate (d).

Figure 5a–d displays the XRD patterns of rCB/ACs prior to the washing treatment, providing a thorough examination of the evolution characteristics of KOH throughout activation. The spectra of the rCB/AC products reveal that the primary phases identified in rCB/ACs are K_2_CO_3_, K_2_CO_3_·1.5H_2_O, K_4_(CO_3_)_2_·(H_2_O)_3_, KHCO_3_, and K_2_O, confirming the presence of carbonates/bicarbonate and oxides as crucial components. According to the existing literature, it is well-acknowledged that carbon elements in precursor materials may initially engage in redox solid–solid interactions, KOH dehydration at 400 °C, and subsequently progress through a solid–liquid state, as detailed in eq 2.^71^ Furthermore, the thermal treatment (<700 °C) might result in an excess of compounds from carbonaceous materials, such as carbon monoxide (CO), hydrogen (H_2_), carbon dioxide (CO_2_), and water vapor (H_2_O) as described in eqs 3–5, supporting previous reported findings.^72^ Ultimately, the procedure can involve metallic potassium obtained from decomposed K_2_O and K_2_CO_3_ at temperatures over 750 °C that intercalates into the carbon matrix and is mainly responsible for generating the pore network (eqs 6 and 7). The initial interaction of K with the C-matrix is followed by many supplementary reactions between KOH and carbon, including distinct active intermediates, as proposed by Otowa et al.^73^
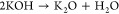
2
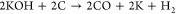
3
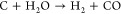
4
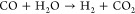
5
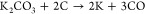
6
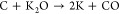
7To delineate the trajectory of chemical evolution more clearly during the KOH treatment of rCB/ACs, the mechanism of rCB activation is outlined based on the applied experimental setup. This confirms and expands on existing proposals in the field as following.

- Multistage transformation to K_2_CO_3_, K_2_CO_3_·1.5H_2_O, and K_4_(CO_3_)_3_·(H_2_O)_3_. The resulting peaks of K_2_CO_3_, K_2_CO_3_·1.5H_2_O, and K_4_(CO_3_)_3_·(H_2_O)_3_ phases for each activation parameters are clearly distinguishable in Figure 5a–d. For all of the examined activation parameters, the reaction might occur at 400 °C with KOH consumption by majority of the carbon atoms in rCB. This leads to a release of H_2_, K, while carbon is oxidized to form carbonates, as presented in eq 8. As a second step, pores are generated through the process of carbon gasification in the presence of CO_2_, effective at temperatures below 700 °C. Concurrently, this phase facilitates the formation of K_2_CO_3_, a reaction notably influenced by the textural properties of the raw material. These properties significantly affect CO_2_ accessibility, underscoring the importance of physical activation (eq 9).^74^ Furthermore, K_2_CO_3_ can undergo a hydration process in a humid environment, leading to the formation of K_2_CO_3_·1.5H_2_O (eq 10). This probably occurs once the rCB/AC samples are taken out from the furnace due to absorption of moisture from the air. The level of hydration is influenced by the surrounding humidity and temperature.
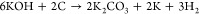
8
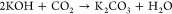
9
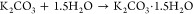
10K_4_(CO_3_)_2_·(H_2_O)_3_, it is most likely produced through a series of reactions involving KOH, CO_2_, and water vapor, under the influence of temperature, KOH concentration, and humidity.
- Decomposition of K_2_CO_3_ to K_2_O. The XRD patterns of rCB/ACs in Figure 5a unequivocally prove the detection of K_2_O within the rCB/AC samples KOH post-thermal treatment at 800–900 °C. The presence of K_2_O, identified in this specific temperature range, provides empirical evidence supporting the breakdown of K_2_CO_3_ into K_2_O and CO/CO_2_. This reaction is a thermally driven process that often occurs at temperatures higher than the standard melting point of K_2_CO_3_ (>700 °C), as shown in eqs 11–13. In addition, the transformation of KOH into K_2_O and H_2_O occurs at elevated temperatures. The presence of other impurities in the activation system can influence the overall process as well, potentially altering the temperature required for effective decomposition. K_2_O can further react with atmospheric CO_2_, leading back to the formation of K_2_CO_3_.
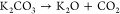
11
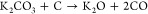
12
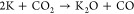
13
- Formation of KHCO_3_

The presence of the KHCO_3_ phase is notably indicated in Figure 5a,b,d. Theoretically, its formation in a reversible reaction and stability can be influenced by the partial pressure of CO_2_ and the moisture content in the environment. Reversibility mainly refers to the ability of KHCO_3_ to undergo decomposition into KOH and CO_2_ (eq 14), resulting in the establishment of a dynamic equilibrium
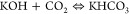
14

### 3.3 Identification of the Synergetic Role of Potassium and Functional Groups (OH^–^, CO_3_^2–^, COOK^–^, C_2_O_4_^2–^, Cl^–^) in Porosity Development

**Figure 6:**
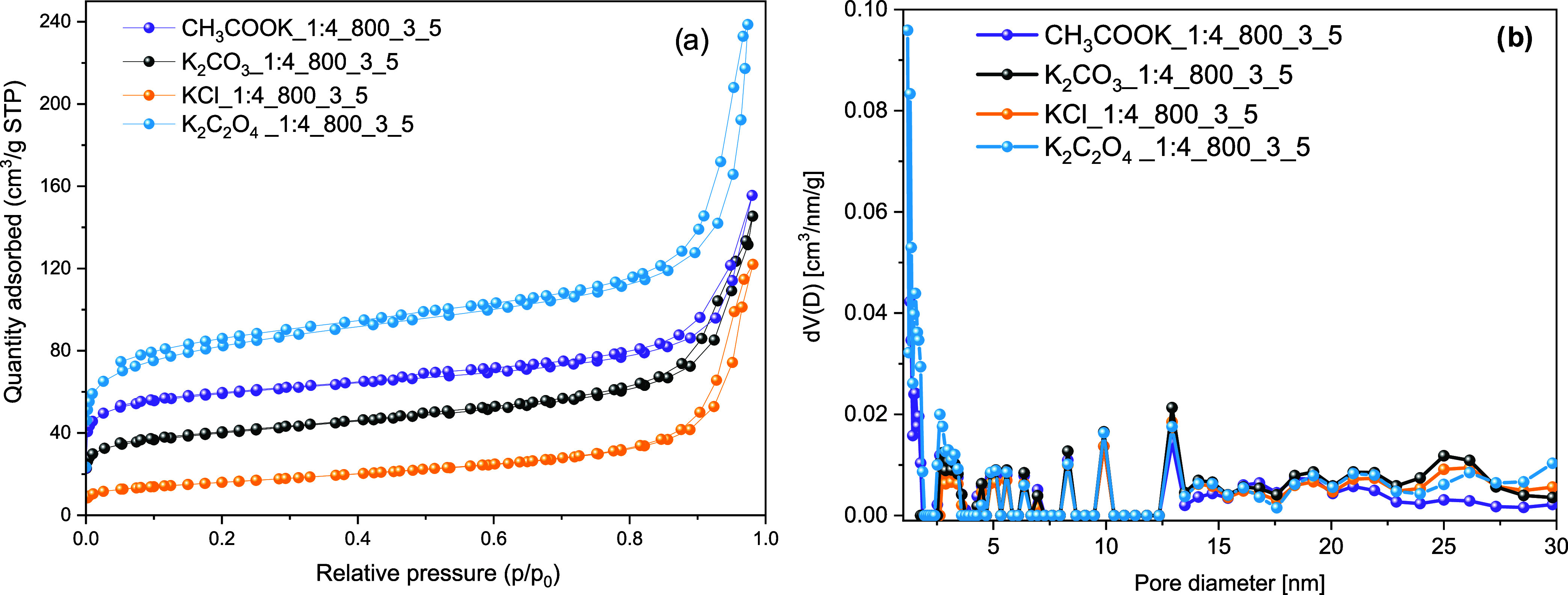
N_2_ adsorption–desorption isotherm curves at 77 K (a) and PSD based on the DFT method on carbon slit pores by the NLDFT model (b) of the rCB/ACs obtained through the utilization of CH_3_COOK, K_2_CO_3_, KCl, and K_2_C_2_O_4_, as potassium-containing activating agents.

Table 7 provides an overview of the textural properties of rCB/ACs activated through different K-containing salts, such as CH_3_COOK, K_2_CO_3_, K_2_C_2_O_4_, and KCl. Despite the KOH_1:4_800_3 sample, K_2_C_2_O_4__1:4_800_3 stood out with the highest BET surface area (299 m^2^/g), total pore volume (0.369 cm^3^/g), and micropore volume (0.093 cm^3^/g) among the samples. On the other hand, KCl_1:4_800_3 exhibited the lowest values among the tested samples, such as 56.7 m^2^/g, 0.189 cm^3^/g, and 0.011 cm^3^/g, for BET, pore volume, and micropore volume, respectively. Moreover, all rCB/ACs share the same type of N_2_ isotherm as KOH, emphasizing uniform adsorption behavior across the studied activators (Figure 6a). It should be noted that their pore size distribution is characterized by a high proportion of mesopores, with clear peaks in size that vary from 2.8 to 13.5 nm, and a broader peak extending up to 30 nm and further to 48 nm (Figure 6b). In addition, rCB/ACs synthesized with potassium oxalate used as an activating agent have exhibited the most developed microporosity among all activators, with the range identified to be between 1.2 and 1.94 nm.

**Table 7:** Textural Properties Associated to rCB/ACs Produced via Chemical Activation with CH_3_COOK, K_2_C_2_O_4_, K_2_CO_3_, and KCl

sample	BET surface area^a^ [m^2^/g]	total pore volume^b^ [cm^3^/g]	micropore volume^c^ [cm^3^/g]	mesopore volume^c^ [cm^3^/g]	micropore volume/total pore volume [%]
KOH_1:4_800_3_5	945 ± 5.6	0.743	0.303	0.440	36.0
CH_3_COOK_1:4_800_3_5	217 ± 2.0	0.240	0.071	0.136	34.3
K_2_CO_3__1:4_800_3_5	146 ± 0.3	0.207	0.043	0.197	17.9
K_2_C_2_O_4_ _1:4_800_3_5	299 ± 1.1	0.369	0.093	0.276	25.2
KCl_1:4_800_3_5	57 ± 0.08	0.189	0.011	0.178	5.8

a Brunauer, Emmett, and Teller method using the Rouquerol criteria.

b *V*_TOT_ calculated by N_2_ adsorption isotherm at a high relative pressure (∼0.99).

c DFT method by the NLDFT model.

**Figure 7:**
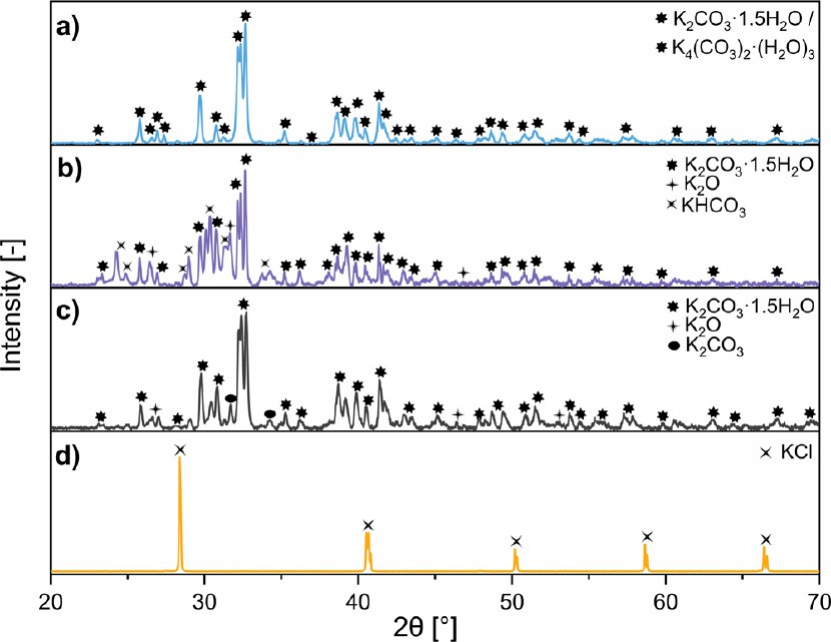
XRD patterns for rCB/ACs before washing treatment produced with CH_3_COOK (a), K_2_C_2_O_4_ (b), K_2_CO_3_ (c), and KCl (d) as potassium-containing activating agents.

By examining the synergetic role of both potassium ions and its related anions (OH^–^, Cl^–^, CO_3_^2–^, C_2_O_4_^2–^, and COOK^–^) in several chemical compounds, it becomes evident that K-based compounds serve as a powerful activator of rCB/ACs. The effectiveness of potassium-containing agents in generating pore structures within a carbon matrix can be influenced by several factors, the types and amounts of gases released during decomposition, the control over the pore generation step, and the specific chemical reactivity of the compound under the activation conditions. Among the four activators, the development of textural properties can be ranked in the following order: KOH > K_2_C_2_O_4_ > CH_3_COOK > K_2_CO_3_ > KCl. The observed findings prove the collaborative specific impact of K and its related anion groups on the formation of a porous structure and intercalated phases during the overall activation reactions, as given in Figure 7.

**Figure 8:**
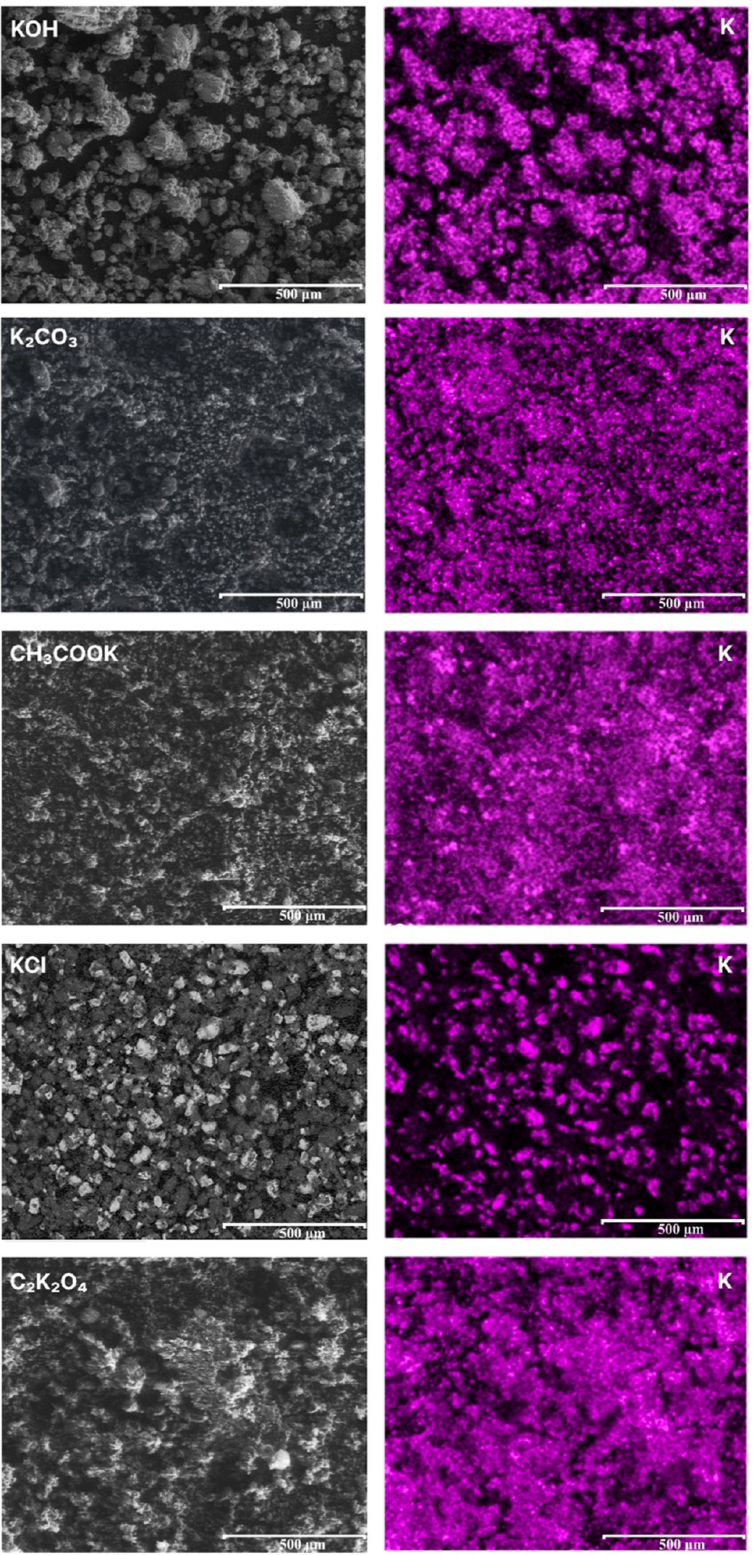
SEM-EDS elemental mapping of potassium for rCB/ACs before washing treatment obtained through KOH, K_2_CO_3_, CH_3_COOK, KCl, and C_2_K_2_O_4_ activation.

Upon reviewing the data of rCB/ACs from Table 7, the efficiency of KOH is clearly noticed, as suggested by the existing literature.^75^ The high molar content of K and the presence of OH^–^ groups are likely responsible for the enhancement of textural characteristics, especially microporosity. The potassium hydroxide undergoes dissociation during high-temperature activation, resulting in the formation of potassium cations (K^+^) and hydroxide (OH^–^) ions. Hydroxyl ions have the ability to chemically attack and weaken the bonds in the carbon substrate, releasing volatile substances, thus efficiently converting the rCB into more porous ACs.^76^ This is also consistent with the quantitative energy-dispersive X-ray spectroscopy analysis (Table 8) and the corresponding SEM-EDS elemental mapping of potassium images (Figure 8). The EDS image of KOH_1:4_800_3_5 shows a high-intensity potassium signal, suggesting a high concentration of K (29.5 wt %) uniformly distributed throughout the rCB/AC sample.

**Table 8:** EDS Semiquantitative Analysis of rCB/AC Samples

sample	K [wt %]	C [wt %]	O [wt %]
KOH_1:4_800_3_5	29.5	31.7	38.8
CH_3_COOK_1:4_800_3_5	20.9	49.8	29.3
K_2_CO_3__1:4_800_3_5	13.1	62.0	24.9
K_2_C_2_O_4_ _1:4_800_3_5	27.5	37.9	34.6
KCl_1:4_800_3_5	19.7	76.0	4.4

In contrast, CH_3_COOK, K_2_CO_3_, and K_2_C_2_O_4_ have lower K/C and K/O ratios. As reported by Yang et al.,^77^ this enables the production of a greater amount of CO_2_ and H_2_O during activation, leading to the formation of larger pores and a decrease in specific surface areas. For K_2_CO_3_ and CH_3_COOK, the EDS mapping of AC/rCB shows a variance in the K distribution, suggesting inhomogeneity due to the local formation of new K-enriched compounds or the dispersal of volatile products. K_2_CO_3_ has lower reactivity toward carbon materials compared to KOH and a much higher melting point. The pure form of K_2_CO_3_ melts at 891 °C. At 800 °C, K_2_CO_3_ is not expected to be completely liquefied but still can soften, releasing a significant amount of CO_2_ (above 650 °C) and K_2_O (below 650 °C), with possible subsequent formation of salt complexes (∼475 °C) on the surface of rCB/ACs (eqs 7–9). On the other hand, the BSE image and EDS map of CH_3_COOK_1:4_800_3_5 show a more heterogeneous distribution of potassium with areas of higher and lower K concentration. Smaller particles and some individual bright spots can be identified, suggesting possible agglomeration or coagulation of potassium-rich phases upon thermal decomposition of CH_3_COOK to K_2_CO_3_ (>303 °C), which undergoes a series of reactions with the carbon structure to produce K-species. In the case of K_2_C_2_O_4_, the EDS mapping reveals a more intense and patchy spread of potassium, indicative of its higher overall presence (27.5 wt %) than the smoother distributions observed for K_2_CO_3_ and CH_3_COOK. Lastly, KCl has been found to be ineffective as an activator, which is primarily attributed to its melting point (∼770 °C), and weak reactivity with rCB, according to the XRD patterns in Figure 7 and SEM-EDS image in Figure 8.

### 3.4 Examining the Phase Impact of KOH Activation: Solid/Liquid State vs Gas State Reactions

**Figure 9:**
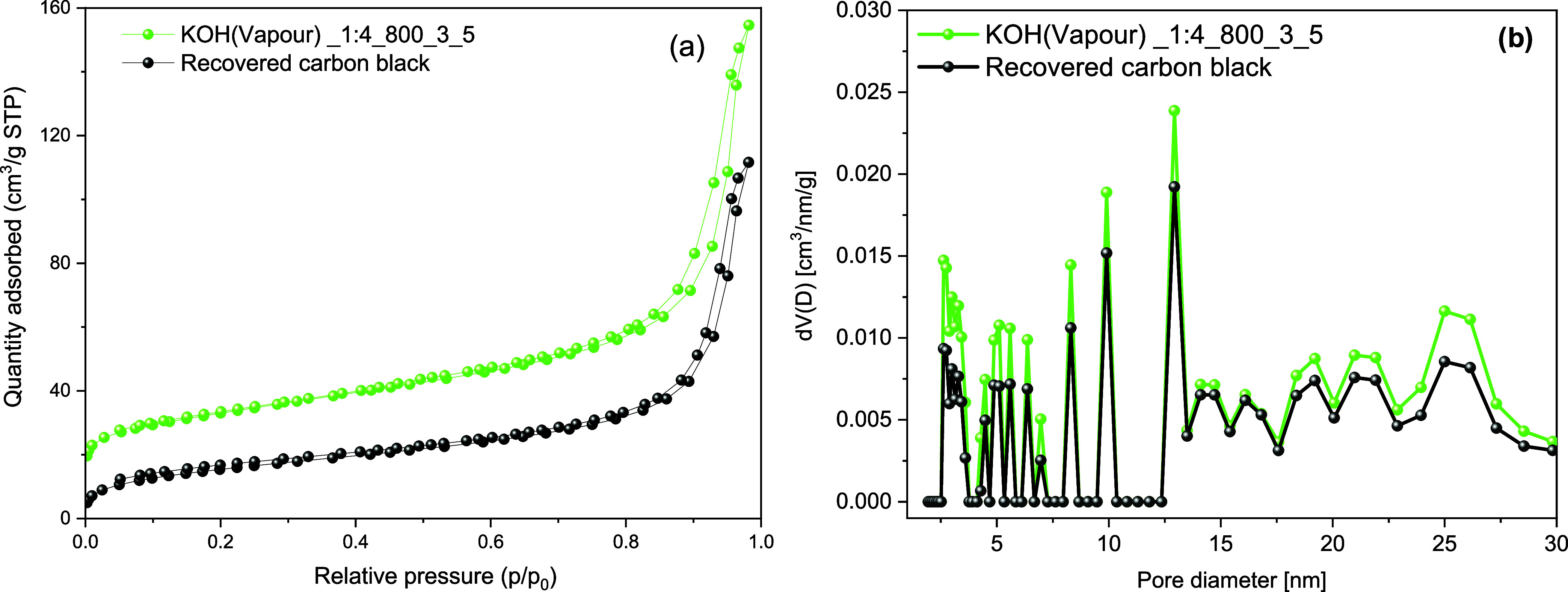
N_2_ adsorption–desorption isotherm curves at 77 K (a) and PSD based on the DFT method on carbon slit pores by the NLDFT model (b) of the rCB and rCB/ACs produced through gas–solid reaction during KOH activation.

The process of activating rCB with KOH requires a complex combination of chemical reactions, including not only the conventional solid–solid (<380 °C) and solid–liquid (≥406 °C) interactions^78^ but also a substantial solid–gas reaction. The use of KOH vapor contributes to the enhancement of the rCB/ACs characteristics, as shown in Table 9 and Figure 9a,b. The gaseous KOH etches the carbon surface and creates pores when the temperature exceeds 600 °C, resulting in the release of gases such as CO_2_ and H_2_O. This phenomenon induces a substantial modification in the surface and pore morphology of the rCB. The *S*_BET_ value increased from 55 m^2^/g to 118, while the *V*_TOT_ increased to 0.239 cm^3^/g. Although the growth of *V*_MIC_ was not significant (0.033 cm^3^/g), the total change in adsorbed quantity of N_2_ and PSD suggests a modification in the texture of the rCB.

**Table 9:** Textural Properties Associated with rCB/ACs Produced through Gas–Solid Reaction during KOH Activation

sample	BET surface area^a^ [m^2^/g]	total pore volume^b^ [cm^3^/g]	micropore volume^c^ [cm^3^/g]	mesopore volume^c^ [cm^3^/g]	micropore volume/total pore volume [%]
rCB	55 ± 0.8	0.170			
KOH(vapor) _1:4_800_3_5	118 ± 0.2	0.239	0.033	0.206	13.8

a Brunauer, Emmett, and Teller method using the Rouquerol criteria.

b *V*_TOT_ calculated by N_2_ adsorption isotherm at a high relative pressure (∼0.99).

c DFT method by the NLDFT model.

**Figure 10:**
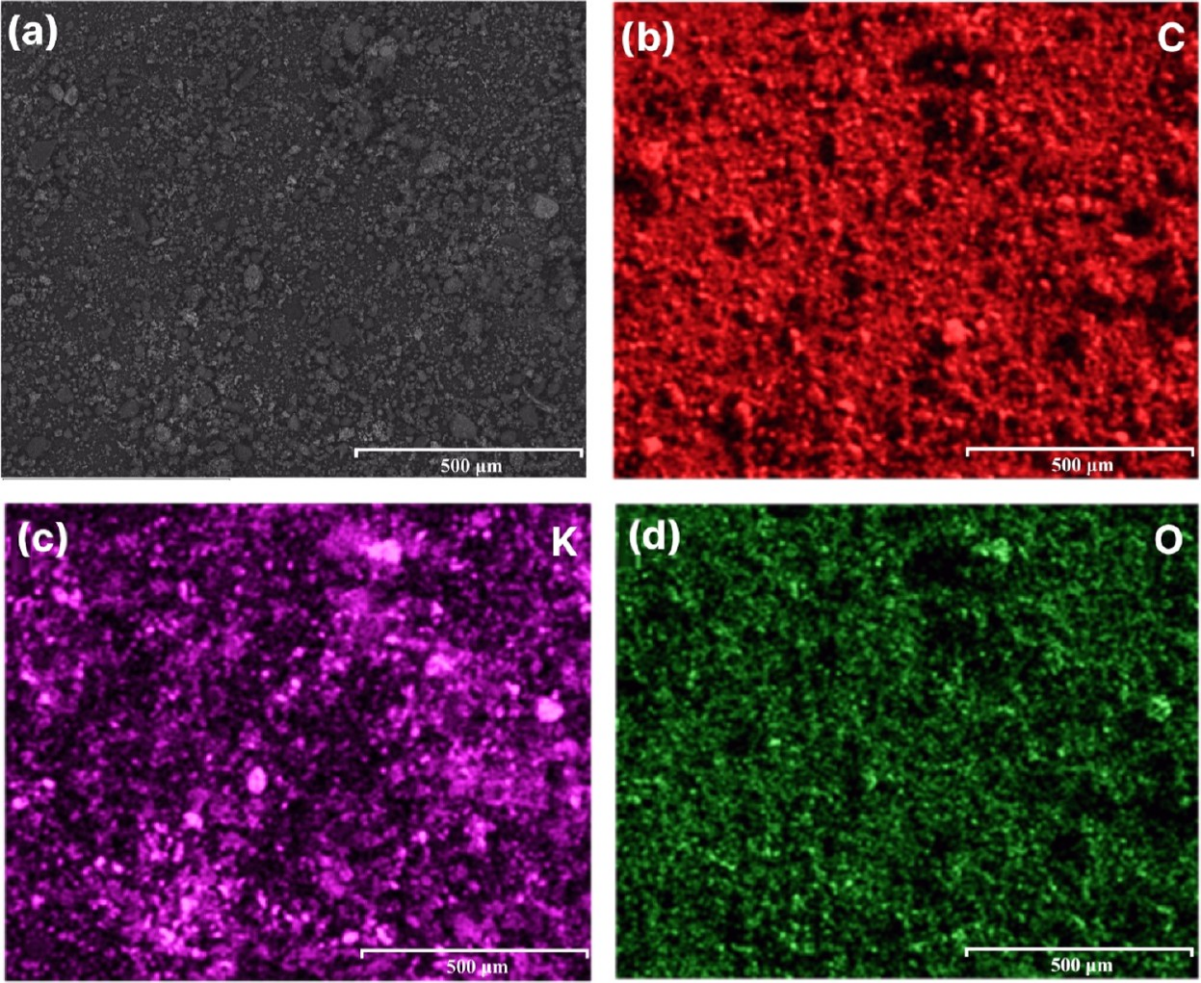
EDS analysis of KOH(vapor)_1:4_800_3_5; (a) representative SEM image and corresponding elemental mapping analysis: C (b), K (c), and O (d).

**Figure 11:**
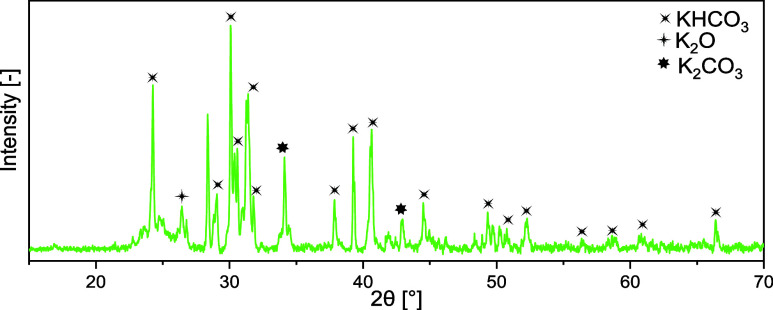
XRD pattern for unwashed rCB/ACs produced through a gas–solid reaction during KOH activation.

The EDS mapping of the rCB sample postreaction with vapor-phase KOH (Figure 10) presents a homogeneous distribution of oxygen, indicating the oxygenation of the carbon structure. Moreover, the rCB/ACs appear to have an irregular K compound distribution from the reaction with vapor-phase KOH. EDS findings directly corroborate with XRD analysis (Figure 11), identifying the formation of KHCO_3_, K_2_O, and K_2_CO_3_ within the sample, which are the expected products of the gas–solid reaction. The presence of KHCO_3_ (60 wt %), K_2_O (10 wt %), and K_2_CO_3_ (30 wt %) in the unwashed rCB/ACs samples indicates that they may be involved in the alterations in textural qualities, including specific surface area and porosity. The formation of KHCO_3_, K_2_O, and K_2_CO_3_ is based on the reaction between gaseous KOH and carbon oxidation. The relative content of C (75 wt %), O (17.8 wt %), and K (7.2 wt %) further supports the impact of the gas–solid reaction on rCB/ACs textural properties.

### 3.5 Comparative Analysis: Potassium (K^+^) vs Sodium (Na^+^) Ions

**Figure 12:**
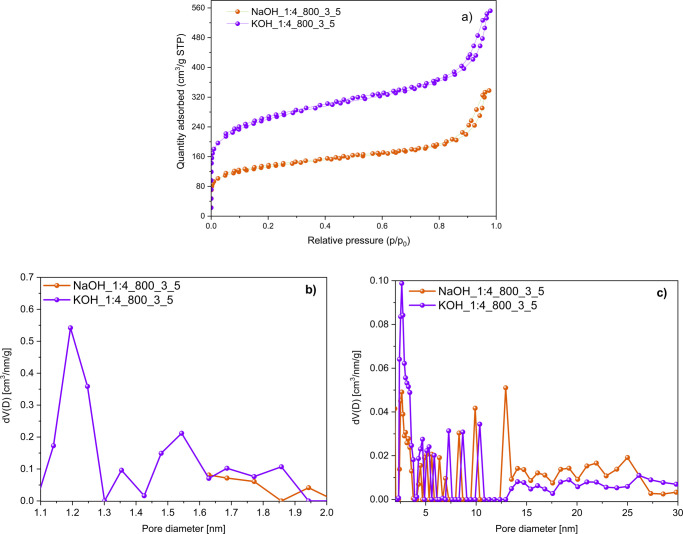
N_2_ adsorption–desorption isotherm curves at 77 K (a), PSD based on DFT method on carbon slit pores by the NLDFT model for the pore diameter in the ranges of 1.1–2 nm (b) and 2–30 nm (c) of the rCB/ACs obtained by KOH/NaOH activation.

The isotherms and textural characteristics presented in Figure 12a and Table 10 indicate that KOH outperforms NaOH in the chemical activation of rCB/ACs. The utilization of NaOH as an activator led to the development of rCB/ACs that possessed *S*_BET_, *V*_TOT_, and *V*_MIC_ of 480 m^2^/g, 0.523 cm^3^/g, and 0.146 cm^3^/g, respectively. Furthermore, the improvement in microporosity (Figure 12b,c) may be attributed to the nature of alkali metal cation (K^+^ vs Na^+^), as studied by Guo et al.^79^ KOH demonstrated a greater propensity to interact with the carbon atoms from rCB, resulting in the formation of micropores in the range of 1.1–1.64 nm and mesopores that vary from 2 to 8.7 nm. On the other hand, NaOH exhibited a greater tendency to generate mesoporosity when the pore width exceeded 9.5 nm. As alkali metal hydroxides intercalate in the carbon network, they function as electron donors during gasification. This property of the hydroxides is most probably responsible for the activation process. Yahya et al.^80^ and Rambabu et al.^81^ have both reported similar findings. Additionally, the intercalation of alkali metals (Na or K) depends upon the crystallinity of the precursor used during the activation procedure, since the structural arrangement, which was formerly overlooked, plays a crucial role. Linares-Solano, Lillo-Ródenas, Marco-Lozar et al.^82^ stated that NaOH is more suitable for carbons with poor ordered atom arrangement (nongraphitic structure), whereas KOH produces better results for those displaying some organizational regularity (graphite-like structure). Based on this understanding, it can be assumed that rCB/ACs tend to have a more crystalline phase than an amorphous one. This makes KOH a preferred choice as an activator due to its effectiveness in engaging with and enhancing the organized, graphite-like arrangement of atoms.

**Table 10:** Textural Properties Associated with rCB/ACs Obtained by NaOH Activation^a^^b^^c^

sample	BET surface area [m^2^/g]	total pore volume [cm^3^/g]	micropore volume [cm^3^/g]	mesopore volume [cm^3^/g]	micropore volume/total pore volume [%]
KOH_1:4_800_3_5	945 ± 5.6	0.743	0.303	0.440	36
NaOH_1:4_800_3_5	480 ± 0.9	0.523	0.146	0.377	28

a Brunauer, Emmett, and Teller method using the Rouquerol criteria.

b *V*_TOT_ calculated by the N_2_ adsorption isotherm at a high relative pressure (∼0.99).

c DFT method by the NLDFT model.

**Figure 13:**
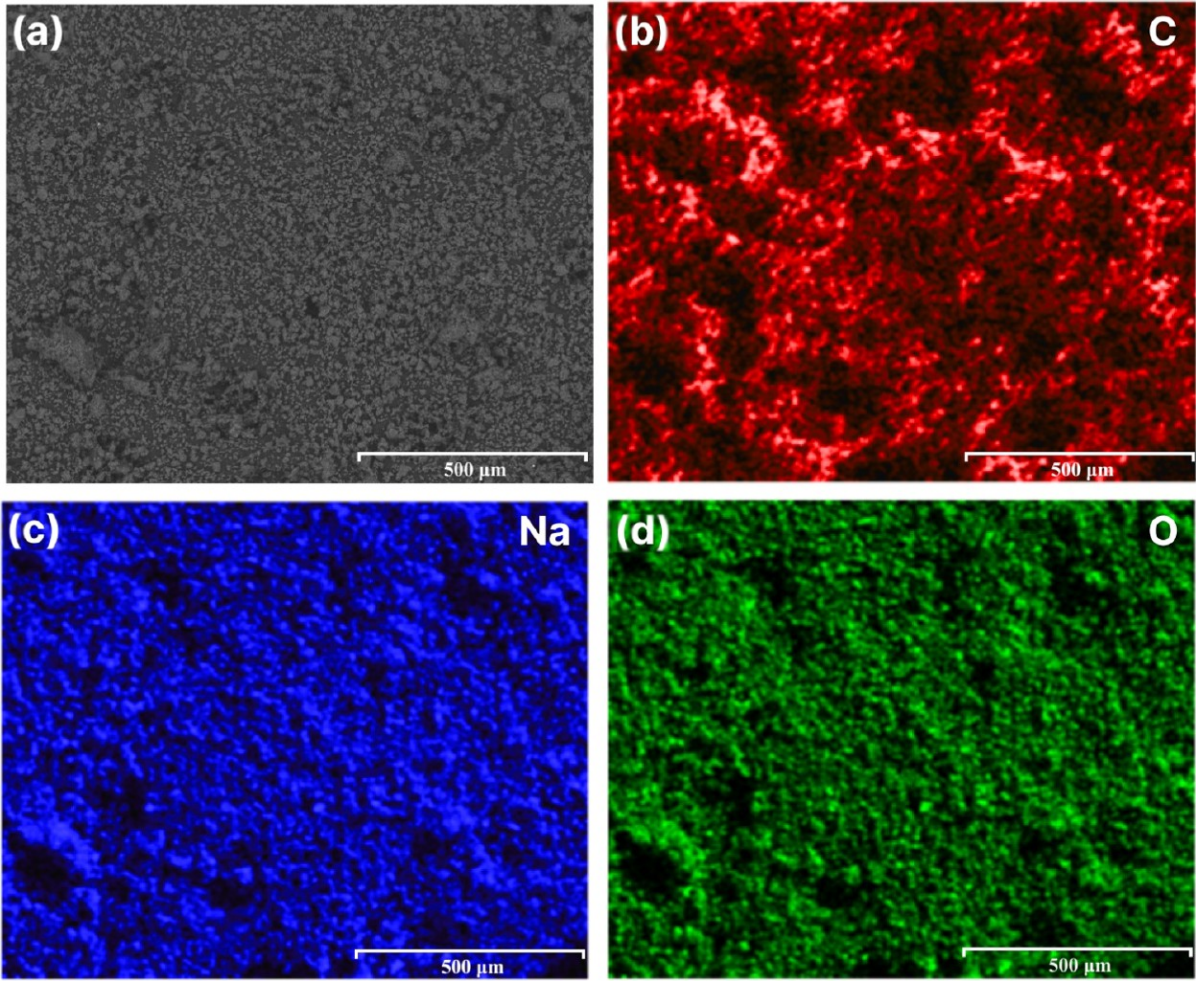
EDS analysis of the NaOH-activated rCB; (a) representative EDS image and corresponding elemental mapping analysis: C (b), Na (c), and O (d).

**Figure 14:**
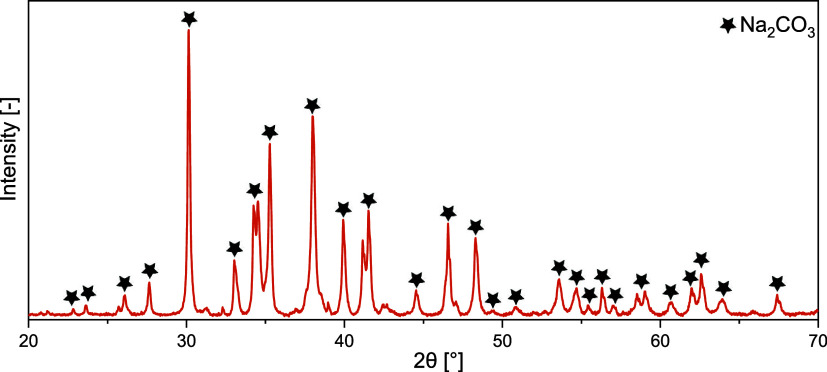
XRD patterns for unwashed rCB/ACs produced through NaOH activation.

The comparison of EDS mapping between NaOH-activated rCB (Figure 13) and KOH-activated rCB (Figure 10) and semiquantitative analysis suggest that NaOH activation results in a lower distribution of alkali metal (Na: 19.8 wt %) compared to KOH (K: 29.5 wt %) but similar oxygen content (O: 37.8 wt % vs O: 38.8 wt %). The XRD analysis presented in Figure 14, which detected only Na_2_CO_3_ as the primary phase in the NaOH-treated rCB/ACs, points to a more limited set of chemical reactions in the case of NaOH compared to that obtained with KOH treatment, where KHCO_3_, K_2_O, and K_2_CO_3_ were identified. This is likely because K^+^, with its larger ionic radius (152 pm) and higher reactivity than Na^+^ (116 pm), is more effective at incorporating in the carbon structure of rCB and reacting with CO_2_. On the contrary, the stronger ionic bonds and lower polarizing power of Na^+^ relative to K^+^ might potentially affect the transport and interaction with the carbon matrix. Therefore, potassium not only promotes more reactions but also leads to more pronounced porosity and surface complexity.

## 4 Conclusions

In this study, the activation mechanism of rCB using potassium-based activators was explored to efficiently transform ELTs into ACs. The objectives were to determine the optimal activation conditions and analyze textural changes, explore the interactions between rCB and potassium alkalis, and unveil a universal process for efficient rCB activation. Techniques such as N_2_ adsorption–desorption at 77 K, XRD, and SEM-EDS were used to investigate the structural and chemical properties of the rCB/ACs. The study identified the below.

- The most effective KOH activation parameters for enhancing the textural properties of ACs were determined, which include an activation temperature of 800 °C, a heating rate of 7 °C/min, a KOH to rCB ratio of 1:5, and a process duration of 4 h. These conditions were found to be optimal for achieving the desired improvements in specific surface area, total pore volume, and micro/mesoporosity.
- XRD patterns of the rCB/ACs products revealed the presence of key phases such as K_2_CO_3_, K_2_CO_3_·1.5H_2_O, K_4_(CO_3_)_2_·(H_2_O)_3_, KHCO_3_, and K_2_O. This confirmed the significant role of carbonates, bicarbonates, and oxides in the activated products, indicating a complex chemical transformation during the activation process. This order of importance was shown to be correlated with the potassium content and its distribution, as revealed using SEM-EDS analysis. The rank of activation potency, from highest to lowest, was as follows: KOH > K_2_C_2_O_4_ > CH_3_COOK > K_2_CO_3_ > KCl.
- The impact of the KOH gas–solid interaction with rCB suggested that improvements are not solely attributed to liquid/solid–solid reactions.
- The KOH activation was primarily attributed to the nature of the alkali metal cation and graphite-like structure of rCB. This emphasizes the importance of the specific chemical characteristics of the activating agent in influencing the final properties of the rCB/ACs.

In essence, the research highlights effective strategies for producing rCB/ACs with superior textural properties, underscoring the significance of carefully chosen activation conditions and the role of potassium-containing activating agents. Moreover, it sets the stage for future studies to explore the potential environmental benefits of utilizing such materials in specific sorption applications, such as water and air purification.
